# Economies of scope in the Norwegian public hospital sector

**DOI:** 10.1007/s10198-024-01704-z

**Published:** 2024-07-18

**Authors:** Nils Arne Lindaas, Kjartan Sarheim Anthun, Sverre A. C. Kittelsen, Jon Magnussen

**Affiliations:** 1 Department of Public Health and Nursing, Norwegian University of Science and Technology, P.O. Box 8905, Trondheim, 7491 Norway; 2 Department of Health Research, SINTEF Digital, P.O. Box 4760, Torgarden, Trondheim, 7465 Norway; 3 Ragnar Frisch Centre for Economic Research, Gaustadalléen 21, Oslo, 0349 Norway; 4 Department of Health Management and Health Economics, University of Oslo, P.O. Box 1089, Blindern, Oslo 0317 Norway

**Keywords:** Economies of scope, Hospital, Reform, New public management, Data envelopment analysis, C14, D24, I18

## Abstract

This study investigates the potential economies of scope in the Norwegian public hospital sector after a major structural and organizational reform. Economies of scope refers to potential cost savings occurring from the scope of production rather than the scale. We use a data driven approach to distinguish between relatively specialized and differentiated hospitals. Using registry data spanning the period 2013–2019, we use non-parametric data envelopment analysis with bootstrapping procedures to investigate the potential presence of economies of scope. This is done separately for three different dimensions of which hospital production can be either specialized or differentiated. The findings suggest that economies of scope are present in the Norwegian hospital sector, meaning that there are cost savings related to the optimal differentiation of the activity. It is difficult to conclude on how these findings relate to the reform.

**Supplementary Information:**

The online version contains supplementary material available at 10.1007/s10198-024-01704-z.

## Introduction

 Norway has a publicly financed national health service (NHS)-type hospital sector. Hospitals are state-owned through four regional health authorities. In this regionalized system two major structural issues are the scale and scope of hospitals. While there is a body of literature discussing economies of scale in hospitals [1], less is known about the potential of economies of scope. Economies of scope is a term from production theory coined by Panzar & Willig [2], which is used to describe situations where cost savings could occur from producing different products in the same rather than separate facilities.

In the hospital sector, the products could be different diagnostic categories, but also types of activities such as elective vs. emergency care, ancillary vs. non-ancillary services or inpatient vs. outpatient treatment. Thus, for policy purposes, the question of economies of scope needs to be related to the specific structural decisions that are to be made. Placing a broad set of hospital functions into the same hospitals can have positive outcomes such as sharing of physical, human, and digital resources across departments during surges of high demand in one department. On the other side, it may have disadvantages related to harmonizing different competence and traditions across departments.

The purpose of this paper is to see whether there is evidence of economies of scope in a regionalized, national health care system such as in Norway. Particularly, we look for economies of scope along the following dimensions:

- Elective vs. emergency care.
- Medical vs. surgical care.
- Inpatient vs. outpatient care.

These three dimensions are chosen because they represent important aspects when determining a regional hospital structure. Elective vs. emergency reflects certainty vs. uncertainty in the level of activity. Whereas the elective treatment can be planned, both in terms of when and where the treatment is to be received, the emergency treatment takes place as quickly as possible and reasonable. While emergency care has fluctuating demand which might be hard(er) to predict, elective care on the other side has predictable demand. The possibility of drawing on resources allocated to planned care would be an advantage for the ability to handle peaks in emergency care demand. A costly alternative would be to have reserve capacity for emergency treatment. On the other hand, during periods of high demand for emergency care, elective care would be disrupted. Kjekshus & Hagen [3] found that ring fencing of elective surgery under certain circumstances had positive effects on hospital efficiency. Also note that economies of scope may depend on economies of scale, i.e., larger emergency care units may have proportionately less uncertainty in demand than smaller ones. Schneider et al. [4] found that hospitals with the highest and lowest average share of emergency activity had the highest efficiency (i.e., a u-shaped relationship).

Patients in need of surgery will require specific resources such as equipment, anesthetics, and surgical theatres. Medical treatment usually consists of (more) nursing-intensive activities, and the patients tend to be older. Since these two ways of treatment usually require such distinct skills and physical resources, the sharing of staff and equipment across departments is not necessarily a viable option during surges of increased demand.

Inpatient vs. outpatient reflects the need for beds. Inpatients spend at least one night at the hospital, while outpatients and day care patients receive treatment and are discharged the same day. Outpatient treatment is considered as being less resource demanding than inpatient treatment, but they often need the same mix of medical expertise. A large share of outpatients is therefore associated with a substantial increase in the number of patients. A higher number of patients can have both positive and negative effects. On one side it can lead to efficiency gains through assembly line-type of organization, but on the other side it can lead to challenges associated with more complex logistics including both staff, patients, and physical resources. Hagen et al. [5] shows that the outsourcing of day-care surgery to for-profit hospitals in Norway leads to lower costs compared to the public hospitals’ costs. Such efficient utilization of resources could indicate diseconomies of scope between outpatient and inpatient services.

If economies of scope are present, then the total costs in the regional hospital sector could be decreased by diversifying or differentiating their production. Conversely, if there are diseconomies of scope the hospital sector could increase their productivity by increasing the degree of specialization. In reality this will be a question of determining a structure that provides a good balance between the different types of activity. An optimal hospital structure should also take into account factors that are not directly related to economies of production, such as patient travel time.

We ask the following research questions, both related to specialization:

- Are there differences in average efficiency between (relatively) specialized and differentiated hospitals?
- Is the Norwegian hospital sector characterized by economies or diseconomies of scope?

**Fig. 1:**
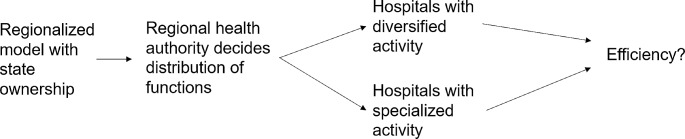
Visual representation of research questions

These questions are analyzed in the theoretical framework of production economics, where the minimum necessary costs of producing a given bundle of health services is represented by the cost function as the frontier of the cost possibility set. Thus, while the first question refers to the distance from the frontier, i.e., how much actual costs exceed necessary costs, the second question refers to the shape of the frontier, i.e., whether total necessary cost of providing health services are higher when provided by differentiated or by specialized hospitals. Figure 1 depicts a simplified visualization of the research questions.

The present organization of the Norwegian hospitals dates back to a hospital ownership reform in 2002. This was a major structural and organizational reform where the ownership of the hospitals was transferred from 19 county councils to the state. The central government decentralized the management of the hospitals to five – later four – regional health authorities (RHA). The reform thus consisted of both centralization and decentralization at the same time [6]. The hospitals were organized as health trusts, being independent legal entities organized under the RHAs. The RHAs have large autonomy when it comes to how services in the health trusts within the RHA should be allocated.

The time-period 2013–2019 is employed mainly for two reasons. First, after the implementation of the 2002 hospital ownership reform, it took several years for the reform to work as intended [7]. During the first decade, the organizational structure was unstable and several mergers took place. The 43 hospitals that were transferred to the state in 2002 had been merged and restructured into 20 hospital trusts by 2013. By investigating the period 2013–2019, we are thus looking at long-term effects of the 2002 reform in a relatively stable period where the changes from the reform have been properly implemented and have had time to settle properly. Second, the data availability for the cost data makes it difficult to extend the time-period further back in time. Cost data is only available for the health trusts, which we will refer to as hospitals, but the health trust may sometimes operate at several geographical locations.

There is a small volume of studies discussing economies of scope in hospitals. However, these studies have investigated economies of scope along different dimensions, which makes it difficult to directly compare studies. Examples of dimensions investigated are inpatient vs. outpatient [8–10], elective vs. emergency [11], acute vs. subacute care [12], intermediate care vs. skilled care [13], as well as different kinds of specialized hospitals vs. generalist hospitals [14–16]. The findings from such studies are difficult to generalize, due to different contexts, methods and findings [16–18]. A large share of the past studies looking at economies of scope are from the United States [8, 12–14, 19, 20]. There are also studies from Europe [10, 11, 15, 16, 21] and Asia [9]. There are large differences between the health systems of the countries covered here, concerning factors such as financing of the health care sector and demographic characteristics of the countries. Studies using parametric frontier methods outnumber those using non-parametric [16]. There are also studies that indirectly investigates economies of scope by comparing specialized and differentiated hospitals, without having economies of scope as the main objective of the study. One example is Berger et al. [22], which uses which uses the Herfindahl-Hirschman index (HHI) to assess hospital specialization.

This study contributes to the field of health services research and health economics by providing an empirical assessment of how a publicly financed health care sector with strong regional autonomy has adapted to reform measures introduced a decade earlier.

## Methods

This study is performed using non-parametric Data Envelopment Analysis (DEA). DEA is a method used to estimate a production possibility frontier (technology) based on the relationship between inputs and outputs in production units [23]. The idea for DEA was first suggested by Farrell [24], and then further developed by Charnes et al. [25]. The initial DEA formulation presented by Charnes et al. assumed constant returns to scale (CRS). This was further extended by Banker et al. [26] to also account for variable returns to scale (VRS). Efficiency measures using VRS efficiencies are generally termed technical efficiency, while the measures using CRS technology are termed technical productivity.

DEA can use either an input or output approach. An input approach estimates how much the inputs can be proportionately reduced while keeping the output constant, while the output approach estimates how much the outputs can be increased while keeping the input constant. Input technical efficiency is defined as the ratio of necessary inputs to actual inputs. Cost functions are defined as the minimum costs necessary to produce a given set of outputs and can easily be calculated with a DEA estimate of technology. In this framework, cost efficiency (CE) is defined as the ratio of necessary to actual costs. In the present analysis, we use an input-approach, with total costs as the only input, and the definitions of cost efficiency and technical efficiency coincide. Cost productivity (CP) can similarly be calculated as the ratio of actual costs to the minimum costs had the unit operated at the optimal scope. The ratio of cost productivity and costs efficiency provides a measure of cost scale efficiency (SE).

Simar & Wilson [27, 28] have shown how one can use bootstrapping to estimate the amount of sampling error in DEA, thus providing a basis for calculating confidence intervals and performing hypothesis tests in DEA models.

DEA can only include inputs and outputs in the production technology specification, and other types of cost shifting variables such as quality, geography etc. can only be included to the extent that they can be formulated as inputs or outputs. The usual workaround is to run a second-stage regression that estimates the multivariate statistical association between the DEA efficiency measures and the external or environmental variables. However, this does not influence the estimates of the shape of the cost frontier, only the interpretation of the efficiency variation. Since the present analysis is primarily concerned with the shape of the frontier, we do not offer a second-stage regression.

The convexity assumption of DEA is problematic since it rules out the possibility of diseconomies of scope on the frontier. The solution we offer is to estimate different cost frontiers for the differentiated and for the specialized hospitals, and to test by the bootstrap method the extent to which these two estimates differ. The use of the distance between two different frontier estimates has been used in scope analyses for hospitals by Kittelsen and Magnussen [21], as well as in other sectors such as water utilities [29] and railways [30], and is closely related to the literature on metafrontiers [31, 32].

We use a data-driven approach to identify “specialized” and “differentiated” hospitals, which we will elaborate in the next section. To answer our first research question, we start off by estimating the efficiency separately for the specialized and the differentiated hospitals, but using a common bootstrap estimate for the cost frontier. For our second research question, we estimate separate best-practice frontiers for the specialized and differentiated hospitals. For each differentiated hospital, we obtain a measure of cost efficiency relative to both frontiers. Our measure of the degree of economies of scope is a cost scope convexity measure (CS), first presented by Kittelsen and Magnussen [21]. They defined the CS as the ratio of the cost efficiency score relative to the specialized and the differentiated frontiers calculated for each of the differentiated hospitals:

$${CS}_{i}= \raisebox{1ex}{${CE}_{i}^{Specialized}$}\!\left/ \!\raisebox{-1ex}{${CE}_{i}^{Differentiated}$}\right.$$

**Fig. 2:**
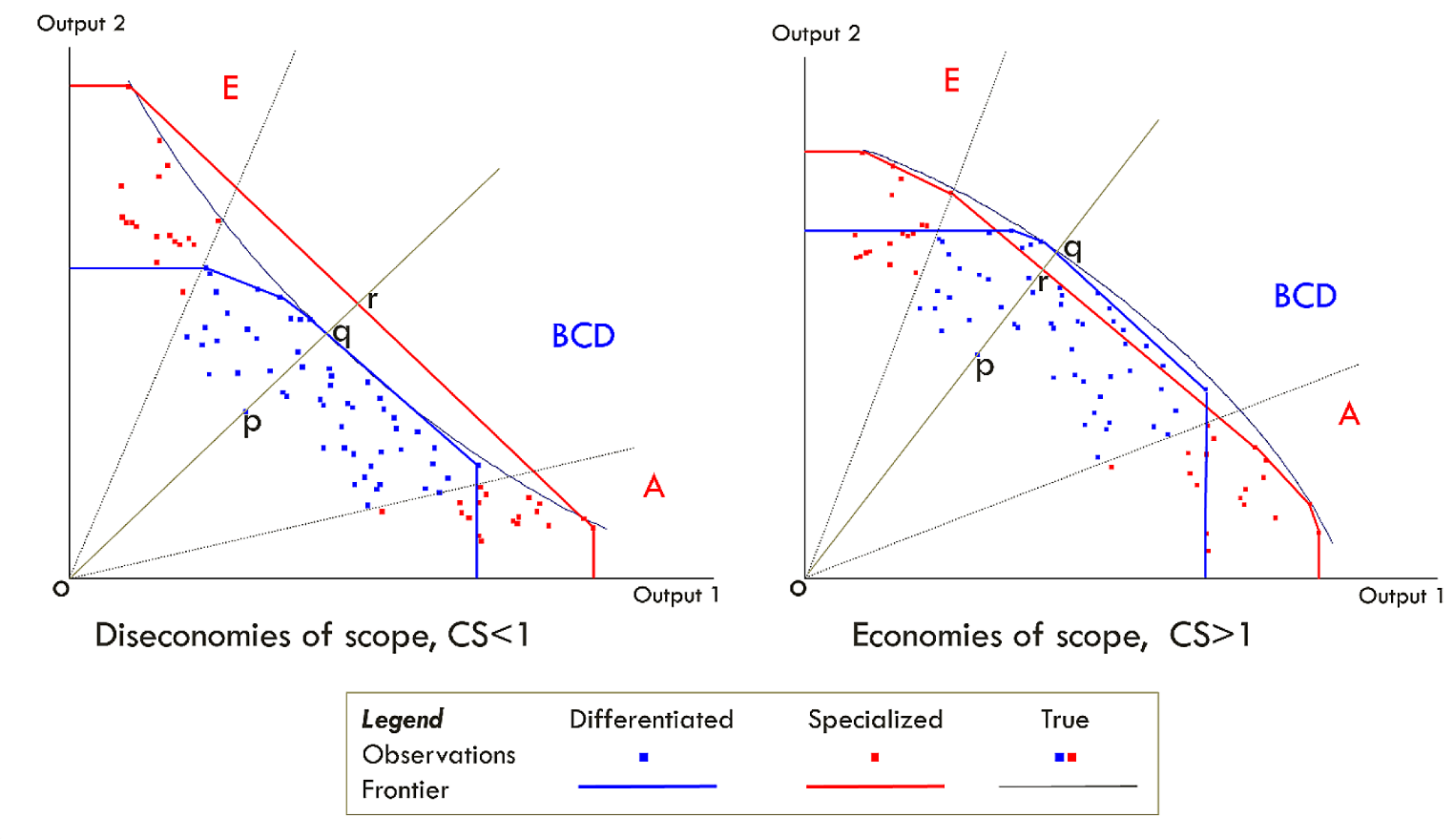
The measure of scope convexity (CS) using the relative distance for two DEA frontier estimates. The curved black line is the true frontier production possibility set for a given cost level. Blue is for the differentiated units in groups BCD and red is for the specialized units in AE. For a differentiated unit at *p*, CS is equal to the distance *oq/or*

This measures the difference in the efficiency frontier of the specialized hospitals and the efficiency frontier from the differentiated hospitals and is illustrated in Fig. 2. If there are economies of scope, the frontier of the differentiated hospitals will lie outside the frontier of the specialized hospitals. The scope convexity measure (CS = *oq/or*) will be greater than one if there are economies of scope, and consequently, if the number is less than one, there are diseconomies of scope. The larger the distance between the frontiers, the larger will be the convexity scope measure, and consequently also the economies of scope. As our test statistic we use the mean of CS across all differentiated hospitals, or alternatively the CS estimate for an average hospital, that is, a hypothetical hospital with average values for inputs and outputs respectively.

### Data

Table 1 shows the descriptive statistics. Hospital outputs are adjusted for case-mix using the DRG (diagnosis-related groups) system. The DRG system provides a reliable classification system for hospital activity which can be used as output measure in efficiency analyses [33]. In this study, we use seven output categories: (1) surgical emergency, (2) medical emergency, (3) surgical elective, (4) medical elective, (5) day-care, (6) outpatient treatment, and (7) all remaining activity. The last group consists of activity that is not easy to fit into the first six groups, for instance chemotherapy and childbirth. We have chosen these output categories, because we believe they can capture key features of hospitals while also allowing for different configurations of hospital activity to be considered efficient. They are inspired by Anthun et al. [33] who used the four categories emergency inpatients, elective inpatients, day-care, and outpatients for their study of productivity growth in the Norwegian hospital sector. In this study, the two first categories are the same as Anthun et al.’s first category, but it is split into surgical and medical. The same applies for the third and fourth category, which is also the same as Anthun et al.’s. These two output categories have been split into two in order to reflect the dimensions we are investigating in this study. Output categories number five and six are identical to the latter two of Anthun et al.’s. This data is collected from the Norwegian Patient Registry. To secure comparability across years, data for all years are grouped into DRG using the same grouper, and aggregated using the same cost-weights (see Anthun et al. [33] for a more thorough elaboration on this). As input, we use sum of total costs, including capital costs. The data on total operating costs are from The Norwegian Directorate of Health, while the data on capital costs are from Statistics Norway. The final dataset is a pooled dataset consisting of repeated observations 19 hospitals over 7 years, thus making a total of 133 observations.

**Table 1:** Descriptive statistics

	All	Dimension					
		Emergency		Outpatient		Surgical	
		Low – A	High – E	Low – A	High – E	Low – A	High – E
	Mean (SD) 133 obs.	Mean (SD) 27 obs.	Mean (SD) 26 obs.	Mean (SD) 27 obs.	Mean (SD) 26 obs.	Mean (SD) 27 obs.	Mean (SD) 26 obs.
**Input (MNOK)**							
Capital and operating costs	4301 (3195)	8644 (4341)	2396 (1270)	3934 (1686)	2587 (1173)	1858 (1065)	8825 (4281)
**Output (DRG points)**							
Surgical emergency	9697 (8139)	20,263 (11,357)	4975 (3267)	8805 (4014)	5278 (3064)	3211 (2198)	21,261 (10,635)
Medical emergency	21,211 (9535)	25,461 (5482)	16,450 (8914)	21,911 (9072)	15,441 (7390)	12,005 (7527)	25,042 (5609)
Surgical elective	13,760 (14,352)	33,742 (20,474)	5164 (3311)	11,516 (6558)	6523 (3339)	3815 (2774)	34,671 (20,159)
Medical elective	5820 (6262)	14,934 (8830)	2336 (1350)	5256 (3120)	2550 (1238)	2098 (1811)	14,714 (9200)
Day-care	3909 (2453)	6651 (3101)	2445 (1438)	3618 (1496)	2502 (1311)	1744 (1182)	6648 (3218)
Outpatient treatment	16,814 (11,808)	31,391 (15,851)	8164 (4250)	12,516 (5545)	12,334 (6225)	6983 (4249)	31,479 (16,198)
Remaining activity	3637 (3103)	7226 (4555)	2277 (1721)	3552 (1940)	1831 (1163)	1449 (1381)	7144 (4726)

We show the correlation between all seven output categories in Table 2. There is a positive correlation between the two surgical categories and the two elective categories. On the other hand, there is a negative correlation between the two medical categories and the two emergency categories.

**Table 2:** Correlations between DRG-points in output categories

	Surgical emergency	Medical emergency	Surgical elective	Medical elective	Day care	Outpatient
Medical emergency	-0.65***					
Surgical elective	0.61***	-0.93***				
Medical elective	0.20**	-0.69***	0.68***			
Day care	-0.35***	0.51***	-0.45***	-0.40***		
Outpatient treatment	-0.20**	-0.11	-0.11	-0.22**	-0.28***	
Rest	0.27***	-0.15*	0.09	0.15*	0.14	-0.49***

Stars indicate significance levels (*** *p* < 0.01, ** *p* < 0.05, * *p* < 0.1)

### Dimensions of specialization

All acute care hospitals are generalist hospitals in the sense that they cover a broad spectrum of services. Thus, no hospital will be fully specialized, and we need another way of distinguishing between hospitals that are “specialized” and “differentiated”. Following Kittelsen & Magnussen [21], we distinguish between different degrees of specialization by first identifying different areas of activity where the hospitals need different treatment technology to operate. Three such areas, or dimensions, are suggested: (1) elective versus emergency care, (2) inpatient versus outpatient treatment, and (3) medical versus surgical patients. The second step is to make a measure of the relative share of activity within the dimension. In the first dimension we look at the relative share of emergency care of the total activity for each hospital. In the second dimension, we measure the relative share of outpatient and day care treatment of the total activity, and in the third dimension we measure the relative share of surgical treatment of the total activity. Third, for each dimension, we sort the pooled dataset over the period 2013–2019 by the size of the relative of share within the dimension, and we divide the sample into five equally sized groups or quintiles. We label the groups from A to E. Group A will have the lowest while group E will have the highest relative share of activity within the chosen dimension. In each dimension, groups A and E thus make up what we will term specialized hospitals. Using elective vs. emergency care as an example, hospitals in group A are specialized in elective care while hospitals in group E are specialized in emergency care. Hospitals in the remaining groups B, C, and D, are classified as differentiated hospitals.

**Fig. 3:**
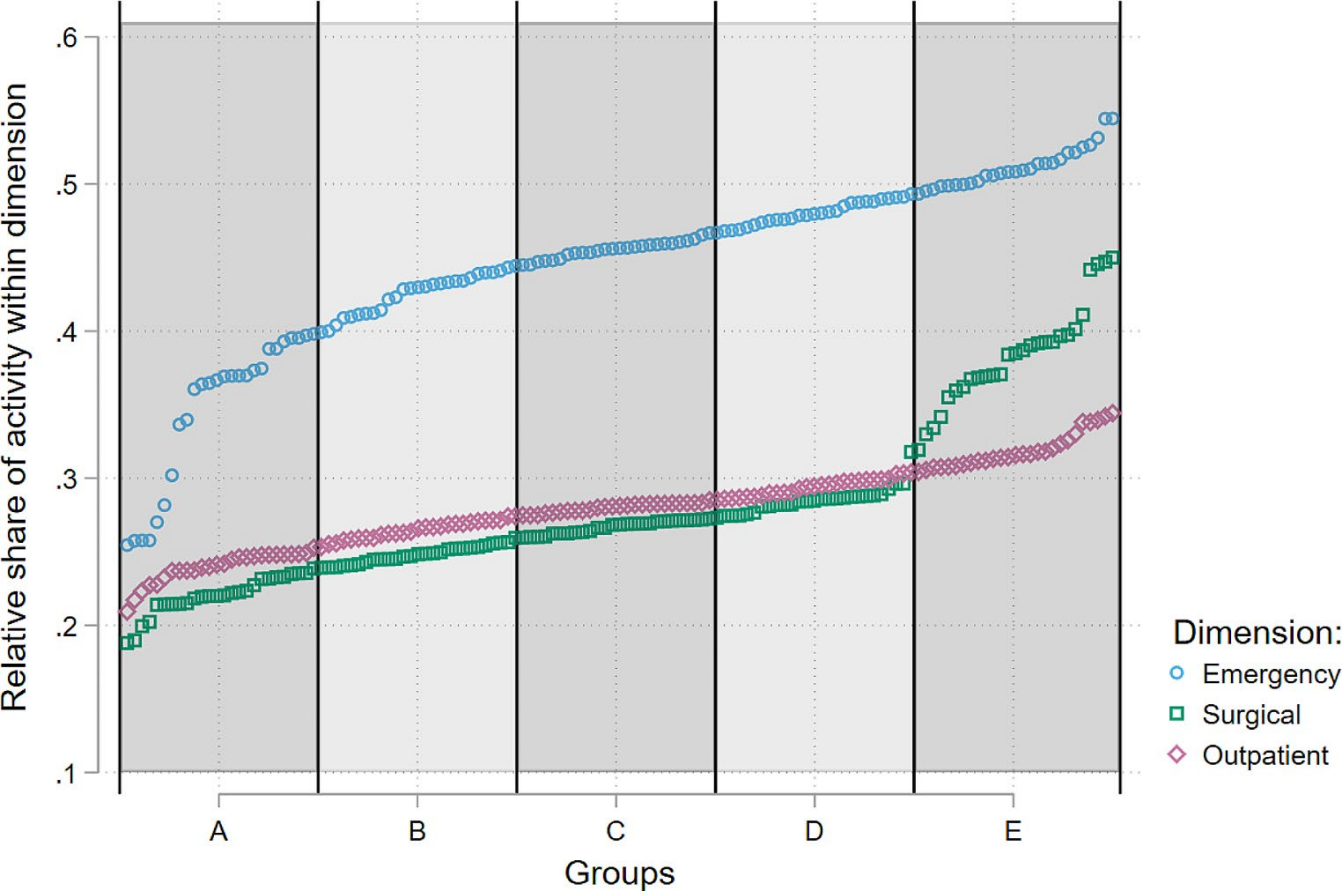
Cut-off point for groups A-E by relative share of activity separately for each dimension

In Fig. 3, we have visualized where the cut-off point is for each dimension. In the elective vs. emergency dimension, group A consists of the hospitals with the lowest relative share of emergency treatment of 25 to 40%, while the share for group E, the hospitals with the highest share of emergency treatment, is between 49 and 55%. In the outpatient versus inpatient dimension, health trusts with the lowest relative share of outpatient activity, group A, have between 21 and 25%, while group E, the hospitals with the highest share of outpatient activity have between 30 and 35%. For surgical versus medical treatment, the hospitals with the lowest relative share of surgical activity, group A, have between 19 and 23%. On the other end group E, the hospitals with the highest share of surgical activity have between 33 and 45%.

From Table 1 we see that the hospitals in the emergency group E are smaller than those in group A. We also see that, measured in *actual* level numbers, the emergency activity in group A is *higher* than in group E, which can seem contradictory. However, since the groups are defined based on relative share, the emergency-activity makes up a larger share of the total activity for the hospital-years in group E compared to group A (as well as the other groups). For the outpatient dimension, we see that the difference between A and E is not as large as for the emergency dimension. The observations in group A are larger here as well, but for the two outpatient variables (day-care and outpatient treatment), the difference is much smaller. For the surgical dimension, we see that the largest observations are in the E group. For the surgical variables, we see that the differences between A and E are substantial.

Since the hospitals are not fully specialized but rather provide broad spectrum of activities, Table 3 also shows the ratio of the activity level between the most and least specialized groups within each dimension. In the elective vs. emergency and inpatient vs. outpatient dimension we see that the relative share of activity in group E is 1.3 times higher than the activity in group A. In the medical vs. surgical dimension, the ratio is somewhat higher with a score of 1.8.

Since we use the pooled dataset when ranking the observations and placing the health trusts to the five intensity groups, it is more precise to refer to the individual observations as hospital-year. When we for each dimension divide the hospital-years into the five intensity groups, we do this independently for each dimension.

**Table 3:** Share in dimensions and ratio between activity in the highest and lowest group

	A (low)	E (high)	Ratio
Share of emergency activity in elective vs. emergency dimension	34.7%	51.2%	1.3
Share of outpatient activity in inpatient vs. outpatient dimension	23.9%	31.9%	1.3
Share of surgical activity in medical vs. surgical dimension	22.0%	38.6%	1.8

## Results

Table 4 shows overall efficiency and productivity estimates for the whole data sample. Looking at the mean non-bootstrapped estimates, we see that they vary between 0.920 for the CRS and 0.960 for the VRS technology. The bias-corrected scores obtained from the bootstrapping process show, as expected, a more conservative estimation. In this case, average technical (cost) efficiency is 0.899 and average technical (cost) productivity is 0.937. Following Farrell’s [24, p. 262] concept of “structural efficiency”, we have also estimated efficiency scores for a hypothetical average unit (AU), which consists of a unit having the input and each output as the average value of all observations. The results for the average unit thus provides a more robust measure than the mean result, thus by definition giving more weight to larger units than smaller units. For the estimation of cost efficiency and cost productivity the average unit score is lower than the mean score, while for cost scale efficiency, the average unit score is higher than the mean score.

**Table 4:** Efficiency measures relative to a common best-practice frontier

	Mean			Average unit		
		Bootstrapped			Bootstrapped	
	Non-bootstrapped estimate	Bias-corrected estimate	Standard error	Non-bootstrapped estimate	Bias-corrected estimate	Standard error
Cost efficiency (CE)	0.960	0.937	0.003	0.897	0.873	0.010
Cost productivity (CP)	0.920	0.899	0.003	0.888	0.862	0.011
Cost scale efficiency (SE)	0.958	0.957	0.002	0.989	0.991	0.006

Scale indicator critical value: 1% (0.982), 5% (0.984), 10% (0.985)

We have tested for CRS vs. VRS, by bootstrapping the cost scale efficiency models with both CRS and VRS under the null hypothesis that the true technology is CRS. The test statistic is the mean estimates cost scale efficiency, corresponding to the test statistic S_1_ in Simar & Wilson [28]. This bias-corrected mean cost scale efficiency with a score of 0.957 is lower than the critical values for this test reported in the bottom of Table 4, and CRS is therefore rejected.

The upper part of Table 5 shows DEA cost efficiency results for both the specialized and differentiated groups as measured against the best practice frontier for the full sample. We include in the lower half the cost scope convexity measure (CS) which are based on separate frontier estimates for the specialized and differentiated groups along each dimension. A more detailed table with estimates for all five subgroups within each dimension (Table S1) and non-bootstrapped CS estimates (Table S2) is included in the Online Resource.

**Table 5:** Efficiency measures for the specialized and differentiated groups within each dimension and cost convex scope measure for each dimension

		Mean cost efficiency		Average unit cost efficiency	
		Boot-strapped estimate	Standard error	Boot-strapped estimate	Standard error
All units		0.937	0.003	0.873	0.010
Elective vs. emergency					
	Specialized (AE)	0.941	0.005	0.874	0.014
	Differentiated (BCD)	0.936	0.003	0.883	0.007
Inpatient vs. outpatient					
	Specialized (AE)	0.939	0.004	0.864	0.006
	Differentiated (BCD)	0.937	0.003	0.884	0.011
Medical vs. surgical					
	Specialized (AE)	0.942*	0.005	0.873	0.014
	Differentiated (BCD)	0.935	0.003	0.891**	0.006
		**Mean cost scope convexity measure**		**Average unit cost scope convexity measure**	
Elective vs. emergency		1.091***	0.008	1.090***	0.011
Inpatient vs. outpatient		1.028***	0.008	1.085***	0.020
Medical vs. surgical		1.109***	0.010	1.117***	0.015

Stars indicate significance levels (*** *p* < 0.01, ** *p* < 0.05, * *p* < 0.1)

The cost efficiency scores are calculated relative to the full sample cost frontier and the significance refers to a score significantly different from the full sample mean

For cost convex scope measure score the significance refers to a score significantly different from 1

The mean const convex scope measure is the mean across all observations in group BCD in each dimension

### Elective versus emergency dimension

The bootstrapped efficiency score for the specialized group (AE) is 0.941 while the score for the differentiated group (BCD) is 0.936, indicating a small but statistically insignificant efficiency advantage for the specialized hospitals. It can be argued that the DEA method makes the extreme observations efficient by default [23]. Thus, using the efficiency measure for the average hospital rather than the average of the efficiency measures will give a more robust score. Here we see that the bootstrapped efficiency score for the average unit is slightly higher for the differentiated group than for the specialized group, and thus opposite of the mean scores. The findings thus indicate that for an average hospital unit, the hospitals with a more equal share between elective and emergency care are more efficient than those with a high share of activity in either. However, these differences are small and not significantly different from the overall mean efficiency.

The CS measure in Table 5 shows a bias-corrected estimate of 1.091. This is significantly higher than 1, which indicates that there are economies of scope in the elective vs. emergency dimension. This means that hospitals can increase their productivity by having a more equal share of elective and emergency activity. As noted above, the CS measure implies that the frontier of the combined differentiated group lies outside the frontier for the combined specialized group. Since this is estimated using costs as the only input, this finding indicates that there are cost savings on the frontier related to the differentiation of these services.

Whereas almost four out of five medical patients are emergency care patients, the majority of surgical patients are elective care patients. Thus, we also did a sub-analysis for the elective vs. emergency dimension, looking separately at medical and surgical patients. This analysis is reported in Table S3 in the Online Resource. The results indicate economies of scope between emergency and elective care within both the medical and surgical subgroups.

### Outpatient versus inpatient treatment

The bias-corrected estimates for the specialized and differentiated groups are approximately equal at 0.939 and 0.937 respectively. Compared to the mean efficiency scores, the score for the average unit shows a difference between the specialized and differentiated groups, where the differentiated group has a higher score than the specialized group. Again, these differences are small and not significant.

The CS measure for this dimension has a bias-corrected estimate of 1.028. Even though this score is lower than the score for the elective vs. emergency dimension, it is still significantly higher than 1, which indicates scope advantages also for the inpatient vs. outpatient dimension. Thus, hospitals can increase their productivity by having a more differentiated share between inpatient and outpatient treatment.

### Surgical versus medical treatment

Looking at the combined specialized and differentiated groups, we see that the specialized group has a higher bias-corrected efficiency score than the differentiated group, with estimates of 0.942 and 0.935 respectively. We see once again that the results for the average unit are opposite from the mean efficiency scores, where the differentiated group has a higher score than the specialized group. In this case the mean differentiated group efficiency is significantly higher than the total average, although the numerical difference is not large.

The bias-corrected CS measure indicates the presence of economies of scope in the medical vs. surgical dimension. This shows that having a more equal share of surgical and medical treatment is preferable to specializing in either direction. The score of 1.109 is significantly higher than 1, and out of the three dimensions tested here, this dimension has the largest score, and therefore has the largest scope advantages associated with increasing the differentiation rather than specializing.

## Discussion

The first research question posed was whether the specialized or differentiated health trusts on average were more efficient. This question is related to the average efficiency scores behind the overall frontier. According to our findings, this question does not have a definitive answer. In all three dimensions the bootstrapped efficiency score is higher for the specialized group. We believe, however, that the measure for the average unit yields a more robust estimate for the efficiency score in this case since the mean score is especially sensitive to extremes in such small sample. The efficiency score for the average unit paints a different picture than the overall mean efficiency score; namely that the differentiated group is more efficient than the specialized group in all three dimensions. All differences are, however, numerically small, and only in one instance significantly different from the overall mean.

Turning to the second question, to what degree we are able to establish (dis)economies of scope along the three dimensions, our findings are clearer. We find indications for scope economies in all three dimensions. We find that for the dimensions elective vs. emergency and medical vs. surgical, the differentiated frontier lies approximately 10% points outside the specialized frontier, which indicates significant economies of scope. For the inpatient vs. outpatient dimension, we also find economies of scope, although substantively lower at 3% points. Although increased specialization in most cases is believed to increase efficiency, we see here that when the hospitals have a large scope of services they must offer, specializing in one direction might come at the cost of productivity since they still must provide a large scope of services.

To assess the practical implication of these findings, we need to look at the cutoff points between the groups within the different dimensions. For the elective vs. emergency dimension, being differentiated refers to having the emergency activity between approximately 40 to 50% of total activity. For medical vs. surgical, being differentiated means having a surgical share of 23–30% of total activity, while for inpatient vs. outpatient, being differentiated means having an outpatient share of 25–30% of total activity. Since we found economies of scope all dimension, this effectively means that hospitals should have at least 40%, but not more than 50% of their activity as emergency. The share of surgical should be between 23 and 30%, and the outpatient share at 25–30%. As we see, the two latter dimensions have a relatively narrow range for what is considered as differentiated. For the inpatient vs. outpatient dimension, it is hard to draw any conclusions of the practical implications, due to the combination of the low value of the cost convexity scope measure and the narrow range for the differentiated group.

Our analysis covers the period from 2013 to 2019, after a period of hospital mergers and a restructuring of the Norwegian hospital sector. A similar analysis has been done by Kittelsen & Magnussen [21], using data from 1992 to 1999. Although there are some differences in the chosen approach (most notably they did not use bootstrapping), the results of the two studies are remarkably similar. This raises the question of whether the reform, being both structural and managerial, has led to a more efficient allocation of tasks between hospitals. In the official reform documents for the 2002 reform, it was stated that it was necessary for the hospitals to have a division of functions that allowed for better efficiency and quality, and to allow units more autonomy and responsibility [34]. The RHAs were given the task of planning the structure, and the New Public Management-inspired reform gave the managers autonomy while keeping the politicians on an “arm’s length” distance when deciding upon the division of functions. The findings from this study suggests that the hospitals can improve efficiency by providing a more even distribution of both elective vs. emergency care and medical vs. surgical care. In both cases using the term “specialized” vs. “differentiated” care signals larger differences that are substantiated by the data. However, as is evident from Fig. 3, for the medical vs. surgical dimensions, there are quite few hospitals that are characterized by a much higher share of surgical patients than the majority of the hospitals. Similarly, there are a number of hospitals with a substantially lower share of emergency care that the majority of hospitals. A starting point would be a more in-depth investigation of these hospitals with the aim of explaining the source of the economies of scope.

By finding economies of scope in the Norwegian hospital sector, this study adds to the list of studies finding economies of scope in hospitals, with studies such as Menke [8], Weaver & Deolalikar [9], Kristensen et al. [10], Freeman et al. [11], Sinay & Campbell [12], Fried et al. [13], Carey et al. [14], Prior & Solà [15], Ferreira et al. [16], Gaynor et al. [20], and Kittelsen & Magnussen [21]. It is not easy to directly compare the results of this study to the results from the international literature, largely due to factors such as large heterogeneity across different health systems and methods applied. Also, the unit of observation differs across studies. Some studies look at the whole sector while others look at certain hospitals within the sector. The dimension inpatient vs. outpatient is perhaps the most common in studies of economies of scope. In our study, this was the lowest degree of economies of scope. Menke [8], Weaver & Deolalikar [9], and Kristensen et al. [10] all found economies of scope along this dimension. However, none of these studies employed non-parametric methods such as DEA, but rather other kinds of cost functions. In our study, we also found economies of scope in the outpatient dimension, but this dimension had the lowest degree of economies of scope.

The original theory of economies of scope, as described by Panzar & Willig [2] referred to the advantages of combining the production of two fully specialized units into one joint unit. Due to the lack of fully specialized hospitals, our definition of economies of scope was based on the difference in the efficiency frontiers of specialized and differentiated hospital units based on a data driven approach to assess relative specialization. Even though the RHAs can to a large degree choose the allocation of services within the region and the hospitals themselves have relatively large autonomy, they still cannot fully choose to specialize within any of the three dimensions tested here. Therefore, the variables used here are not fully independent of each other. A consequence of this is that the findings of economies of scope might be partly driven by the fact that hospitals cannot fully choose how to allocate their services. However, even if hospitals aren’t completely free to choose their service mix, we believe that understanding the efficiency implications of their current service mix still can provide valuable insights for policymakers and hospital managers.

There are some methodological limitations with this study that need to be addressed. Concerning the grouping of activity, some of the groups that have been combined in this study have different underlying production functions. For instance, within elective treatment, the production functions for medical and surgical patients are different. Optimally the inpatient treatment group should also have included outpatient procedures that were conducted as a necessary preceding or follow-up to the inpatient treatment. This was, however, not possible to do with the data at hand. This is the reason for why we in this study investigate economies of scope along three dimensions, rather than just one. Since we employ a pooled dataset, we are comparing hospitals from one time period to hospitals from another time period. For instance, for the inpatient vs. outpatient dimension, there has been a shift over time with a substantial increase in the volume of outpatient treatment. The best-practice frontier technology may not have been much affected since previous studies have found low rates of technical change/frontier shift [33]. Another relevant question is whether we are confusing economies of scale and scope. Since we are using a scope measure defined by VRS fronter estimates and additionally analyzing the specialized groups A and E *combined*, these problems should to some degree be reduced. The only dimension in which we see a substantial difference in mean efficiency between group A and E is in the medical vs. surgical dimension (table S1 in Online Resource).

The inability of DEA to include other cost shifters than inputs or outputs is a limitation of the analysis. Another issue related to the DEA methodology is that some observations are efficient by default. Since we have relatively few observations in this study, our findings are possibly more prone to this problem. By using the measure of the average unit, we believe that we have used a more robust measure for efficiency scores across the specialized and differentiated hospitals. It is further a positive sign that the mean score and the average unit score are similar for the two dimensions with the highest degree of economies of scope (elective vs. emergency and medical vs. surgical).

In this study we use hospital data aggregated to the health trust level, as this is the lowest possible level with reliable data. We do not know how costs are allocated between different locations or departments, and whether any specific specialization advantages could be reaped at the within-trust level. As pointed out by Kristensen et al. [10], by using such highly aggregated data, there might be instances where some specific hospital services faces economies of scope, and others face diseconomies of scope, which we are not able to capture. This means that the findings from this study should not be used to take policy decisions at lower levels but rather to inform about overall sector wide trends for the three dimensions. In addition, health trusts that within our definition are specialized in one direction in one or more dimensions cannot decide to become differentiated from one day to the other. Rather, they can use these findings when planning future activity, investments, and personnel strategies. There are, however, some policy implication of this study on a higher level. The fact that we identified economies of scope should be of interest for the politicians and policy makers, since this indicates that a (relatively) even spread of activity is associated with lower costs. Planning for potential future reforms and structural changes of the sector should therefore take this into account.

In this study we tried to further investigate whether the results in the elective vs. emergency dimension were driven by either medical or surgical patients, but these analyses did not yield any clear results. Future research should therefore focus revealing which factors within each dimension that contribute to these results. Further, future research should also include qualitative studies investigating how, why, and to what degree hospital managers make decisions concerning the specialization and diversification of activity. This would help increase the knowledge of this topic from a policy perspective.

In conclusion, our findings suggest that economies of scope are present in the Norwegian hospital sector. Although this study has taken place during a relatively stable period, where the 2002 hospital ownership reform measures have settled, we cannot conclude with certainty that this present situation is a direct effect of the reform, or whether it is a result of pragmatic developments over time.

## Electronic supplementary material

Below is the link to the electronic supplementary material.

Supplementary Material 1
