# Supplementary material for: Economies of scope in the Norwegian public hospital sector
Source: Eur J Health Econ. 2024 Jul 18;26(2):325–35. doi: 10.1007/s10198-024-01704-z (PMC11889039; doi:10.1007/s10198-024-01704-z)
Supplement: Supplementary file 1 — Supplementary Material 1 [file 10198_2024_1704_MOESM1_ESM.docx]

Paper 3 Supplementary Information (SI)

# Tables:

Table S1: DEA results for all groups and subgroups using variable returns to scale with an input-orientation.

|  |  |  | Mean cost efficiency | | |  | Average unit cost efficiency | | | |
| --- | --- | --- | --- | --- | --- | --- | --- | --- | --- | --- |
|  |  |  |  | Bootstrapped | |  |  | Bootstrapped | | |
|  |  |  | Non-bootstrapped estimate | Bias-corrected estimate | Standard error |  | Non-bootstrapped estimate | Bias-corrected estimate | Standard error | |
| All units |  |  | 0.960 | 0.937 | 0.003 |  | 0.897 | 0.873 | 0.010 | |
| Elective vs emergency | | | | | | | | | |  |
| Specialized (AE) | | | 0.968 | 0.941 | 0.005 |  | 0.904 | 0.874 | 0.014 | |
|  | A |  | 0.968 | 0.936 | 0.008 |  | 0.929 | 0.902*** | 0.012 | |
|  | E |  | 0.968 | 0.946*** | 0.005 |  | 0.910 | 0.887 | 0.010 | |
| Differentiated (BCD) | | | 0.956 | 0.936 | 0.003 |  | 0.901 | 0.883 | 0.007 | |
|  | B |  | 0.934 | 0.917*** | 0.003 |  | 0.861 | 0.841*** | 0.008 | |
|  | C |  | 0.976 | 0.951*** | 0.005 |  | 0.946 | 0.926*** | 0.006 | |
|  | D |  | 0.960 | 0.940 | 0.004 |  | 0.906 | 0.887 | 0.006 | |
| Inpatient vs outpatient | | | | | | | | | |  |
| Specialized (AE) | | | 0.961 | 0.939 | 0.004 |  | 0.884 | 0.864 | 0.006 | |
|  | A |  | 0.960 | 0.934 | 0.005 |  | 0.904 | 0.883 | 0.008 | |
|  | E |  | 0.963 | 0.943*** | 0.005 |  | 0.898 | 0.873 | 0.016 | |
| Differentiated (BCD) | | | 0.961 | 0.937 | 0.003 |  | 0.909 | 0.884 | 0.011 | |
|  | B |  | 0.954 | 0.931 | 0.005 |  | 0.892 | 0.866 | 0.012 | |
|  | C |  | 0.974 | 0.947*** | 0.006 |  | 0.942 | 0.918*** | 0.009 | |
|  | D |  | 0.955 | 0.933 | 0.005 |  | 0.906 | 0.884 | 0.007 | |
| Medical vs surgical | | | | | | | | | |  |
| Specialized (AE) | | | 0.967 | 0.942* | 0.005 |  | 0.901 | 0.873 | 0.014 | |
|  | A |  | 0.975 | 0.953*** | 0.005 |  | 0.913 | 0.889* | 0.008 | |
|  | E |  | 0.959 | 0.930 | 0.008 |  | 0.925 | 0.899*** | 0.014 | |
| Differentiated (BCD) | | | 0.957 | 0.935 | 0.003 |  | 0.910 | 0.891** | 0.006 | |
|  | B |  | 0.951 | 0.932 | 0.004 |  | 0.891 | 0.870 | 0.009 | |
|  | C |  | 0.952 | 0.930 | 0.005 |  | 0.911 | 0.891** | 0.006 | |
|  | D |  | 0.969 | 0.944*** | 0.005 |  | 0.937 | 0.916*** | 0.006 | |

Stars indicate significance levels (*** p<0.01, ** p<0.05, * p<0.1).
Significance refers to a score significantly different from the full sample.

Table S2: Convexity scope measure with non-bootstrapped estimates.

|  |  | Mean | | | |  | Average unit | | | |  |
| --- | --- | --- | --- | --- | --- | --- | --- | --- | --- | --- | --- |
|  |  |  | Bootstrapped | | |  |  | Bootstrapped | | |  |
|  |  | Non-bootstrapped estimate | Bias-corrected estimate |  | Standard error |  | Non-bootstrapped estimate | Bias-corrected estimate |  | Standard error | |
| Cost convex scope measure | |  |  |  |  |  |  |  |  |  | |
|  | Elective vs emergency | 1.090 | 1.091*** |  | 0.008 |  | 1.092 | 1.090*** |  | 0.011 | |
|  | Inpatient vs outpatient | 1.026 | 1.028*** |  | 0.008 |  | 1.085 | 1.085*** |  | 0.020 | |
|  | Medical vs surgical | 1.105 | 1.109*** |  | 0.010 |  | 1.111 | 1.117*** |  | 0.015 | |

Stars indicate significance levels (*** p<0.01, ** p<0.05, * p<0.1).
The significance score refers to a score significantly different from 1.
The mean const convex scope measure is the mean across all observations in group BCD in each dimension.

Table S3: Convexity scope measure separately for medical and surgical patients within elective vs emergency dimension.

|  |  | Mean | | | |  | Average unit | | | |  |
| --- | --- | --- | --- | --- | --- | --- | --- | --- | --- | --- | --- |
|  |  |  | Bootstrapped | | |  |  | Bootstrapped | | |  |
|  |  | Non-bootstrapped estimate | Bias-corrected estimate |  | Standard error |  | Non-bootstrapped estimate | Bias-corrected estimate |  | Standard error | |
| Cost convex scope measure | |  |  |  |  |  |  |  |  |  | |
|  | Medical elective vs medical emergency | 1.041 | 1.047*** |  | 0.009 |  | 1.033 | 1.038*** |  | 0.011 | |
|  | Surgical elective vs emergency | 1.049 | 1.042*** |  | 0.006 |  | 1.035 | 1.026* |  | 0.013 | |
|  |  |  |  |  |  |  |  |  |  |  | |

Stars indicate significance levels (*** p<0.01, ** p<0.05, * p<0.1).
The significance score refers to a score significantly different from 1.
The mean const convex scope measure is the mean across all observations in group BCD in each dimension.

# Technical details

The DEA method estimates the production possibility set or technology $\hat{T}$ from observations of output vectors and input vectors $(y^{j},x^{j})$ for each hospital $j\in N$. By defining groups of hospitals $G\subseteq N$ one can express the estimate of the production set of each group as

$\hat{T}^{G}=\left\{\left(\mathbf{x,y}\right)\left|\mathbf{y}\leq\sum_{j\in G} \lambda_{j}\mathbf{y}^{j}\mathbf{,x}\geq\sum_{j\in G} \lambda_{j}\mathbf{x}^{j},\sum_{j\in G} \lambda_{j}=1\right.\right\}$ (1)

The Cost function or frontier reflecting minimum necessary costs $C$ for group $G$ observations, as well as Cost efficiency $CE$ and Cost productivity $CP$ for any hospital $i$, which need not belong to group $G$*,* can following Farrell (1957) then be estimated as

$\begin{matrix} \mathrm{C}(\mathbf{w}^{i},\mathbf{y}^{i})=\mathrm{Min}_{\mathbf{x}}\left\{\sum_{k} w_{k}^{i}x_{k}\left|(\mathbf{x,y}^{i})\in\hat{T}^{G}\right.\right\} \\ CE_{i}^{G}=\frac{\mathrm{C}(\mathbf{w}^{i},\mathbf{y}^{i})}{\sum_{k} w_{k}^{i}x_{k}^{i}}=\mathrm{Min}_{\mathbf{x}}\left\{\frac{\sum_{k} w_{k}^{i}x_{k}}{\sum_{k} w_{k}^{i}x_{k}^{i}}\left|(\mathbf{x,y}^{i})\in\hat{T}^{G}\right.\right\} \\ CP_{i}^{G}=\mathrm{Min}_{\mathbf{x},\theta}\left\{\frac{\sum_{k} w_{k}^{i}x_{k}}{\sum_{k} w_{k}^{i}x_{k}^{i}}\left|(\theta\mathbf{x,}\theta\mathbf{y}^{i})\in\hat{T}^{G}\right.\right\} \end{matrix}$ (2)

Scale efficiency $SE$ can further be defined by

$SE_{i}^{G}=\frac{CP_{i}^{G}}{CE_{i}^{G}}$ (3)

For the main results, $G$ includes all hospital observations. The scope convexity measure, which expresses how much the frontier estimated from differentiated units in BCD is more productive (>1) or less productive (<1) than the frontier defined from the specialized units in AE, can for each hospital $i$ be defined as

$CS_{i}=\frac{CE_{i}^{AE}}{CE_{i}^{BCD}}$ (4)

Hospital $i$ can belong to either (or neither) group, so that the distance between frontiers is measured in the direction in output space that corresponds to the output mix of hospital $i$. The scope convexity measure will only be relevant for the differentiated hospitals in BCD when the question is whether these would be more productive if they remained differentiated than if the same product mix was produced separately in specialized hospitals of the same approximate scale.
